# Antiviral activities of plant-derived indole and β-carboline alkaloids against human and avian influenza viruses

**DOI:** 10.1038/s41598-023-27954-0

**Published:** 2023-01-28

**Authors:** Akram Hegazy, Sara H. Mahmoud, Yaseen A. M. M. Elshaier, Noura M. Abo Shama, Nasr Fawzy Nasr, M. A. Ali, Assem Mohamed El-Shazly, Islam Mostafa, Ahmed Mostafa

**Affiliations:** 1 Department of Agricultural Microbiology, Faculty of Agriculture, Cairo University, Giza District, 12613 Giza Egypt; 2 Center of Scientific Excellence for Influenza Viruses, National Research Centre, Giza, 12622 Egypt; 3 Department of Organic and Medicinal Chemistry, Faculty of Pharmacy, University of Sadat City, Sadat City, 32897 Menoufia Egypt; 4 Department of Pharmacognosy, Faculty of Pharmacy, Zagazig University, Zagazig, 44519 Sharkia Egypt; 5 Faculty of Pharmacy, El Saleheya El Gadida University, El Saleheya El Gadida, 44813 Sharkia Egypt

**Keywords:** Drug discovery, Microbiology

## Abstract

The persistent evolution of drug-resistant influenza strains represents a global concern. The innovation of new treatment approaches through drug screening strategies and investigating the antiviral potential of bioactive natural-based chemicals may address the issue. Herein, we screened the anti-influenza efficacy of some biologically active indole and β-carboline (βC) indole alkaloids against two different influenza A viruses (IAV) with varied host range ranges; seasonal influenza A/Egypt/NRC098/2019(H1N1) and avian influenza A/chicken/Egypt/N12640A/2016(H5N1). All compounds were first assessed for their half-maximal cytotoxic concentration (CC_50_) in MDCK cells and half-maximal inhibitory concentrations (IC_50_) against influenza A/H5N1. Intriguingly, Strychnine sulfate, Harmalol, Harmane, and Harmaline showed robust anti-H5N1 activities with IC_50_ values of 11.85, 0.02, 0.023, and 3.42 µg/ml, respectively, as compared to zanamivir and amantadine as control drugs (IC_50_ = 0.079 µg/ml and 17.59 µg/ml, respectively). The efficacy of the predefined phytochemicals was further confirmed against influenza A/H1N1 and they displayed potent anti-H1N1 activities compared to reference drugs. Based on SI values, the highly promising compounds were then evaluated for antiviral efficacy through plaque reduction assay and consistently they revealed high viral inhibition percentages at non-toxic concentrations. By studying the modes of antiviral action, Harmane and Harmalol could suppress viral infection via interfering mainly with the viral replication of the influenza A/H5N1 virus, whilst Harmaline exhibited a viricidal effect against the influenza A/H5N1 virus. Whereas, Strychnine sulfate elucidated its anti-influenza potency by interfering with viral adsorption into MDCK cells. Consistently, chemoinformatic studies showed that all studied phytochemicals illustrated HB formations with essential peptide cleft through the NH of indole moiety. Among active alkaloids, harmalol displayed the best lipophilicity metrics including ligand efficiency (LE) and ligand lipophilic efficiency (LLE) for both viruses. Compounds geometry and their ability to participate in HB formation are very crucial.

## Introduction

Over the last century, the pandemic consequences of influenza A viruses (IAV) are more hazardous and frequent, even when compared with the morbidity and mortality rates of the current devastating global pandemic of COVID-19^1,2^.

The Influenza viruses are belonging to the Orthomyxoviridae family and are classified into 4 genera: influenza A (IAV), influenza B (IBV), influenza C (ICV), and influenza D (IDV) viruses. All reported occasional influenza pandemics and seasonal epidemics are caused mainly by IAV infections^1,3^. The evolution of pandemic IAV variants occurs commonly via the genetic exchange between viral RNA segments of different subtypes of IAVs in a genomic phenomenon called “antigenic shift or reassortment”. Whereas, the evolution of endemic IAV variants occurs via a continuous change in the viral RNA genome during viral replication “antigenic drift” in different hosts^1,4^.

The innovation of antivirals has long paved the way for treating influenza in developing and developed countries. However, the future efficacy of the commercially available antivirals is somewhat suspicious because viruses are developing resistance in an inevitable way to the already established antiviral drugs. Plants are still the best choice for extracting and isolating naturally-based antiviral agents^5^.

Alkaloids are belonging to a major chemical group that is found in microbes, plants, and animals^6^. These small cyclic nitrogenous compounds are alkaline in nature, hence the name, and they are found in 20% of plants^7^. They are categorized into indole, quinoline, isoquinoline, tropane, steroidal, pyridine, and pyrrolizidine based on their molecular structure^8^. Indole alkaloids class are those alkaloids with an organic structure consisting of a pyrrole linked to a benzene ring. Such compounds are produced from plants as secondary metabolites, and they exhibited antiviral activities against a wide variety of viruses such as IAV^9^, hepatitis C virus (HCV)^10^, human immunodeficiency virus-1 (HIV-1)^11^, dengue virus (DENV)^12^, zika virus (ZIKV) and chikungunya virus (CHIKV)^13^ and may play a key role in combating the newly emerged coronavirus; severe acute respiratory syndrome coronavirus-2 (SARS-CoV-2)^14–16^. The aromatic compounds: β-carbolines (naturally and/or synthetically indole alkaloid derivatives) possess numerous biological activities including antiviral activities against different emerging and reemerging viruses such as herpes simplex virus-1 (HSV-2), DENV-2, and enterovirus-71 (EV-71), HIV and Poliovirus (PV)^17–20^. The β-carbolines are common in *Peganum harmala*, this plant is common in Egypt. In folk medicine, it is used as an analgesic, antiseptic, and emmenagogue and in the treatment of asthma, colic, jaundice, and lumbago^21^.

Herein, we investigated the antiviral potential of some purified naturally derived indole and β-carboline indole alkaloids against avian and human influenza A/H5N1 and A/H1N1viruses, respectively. The virtual mode of action for the biologically active alkaloids was also provided.

## Materials and methods

### Cell lines and viruses

Madin Darby Canine Kidney (MDCK) cells were kindly obtained from the cell culture collections of the Centre of Scientific Excellence for Influenza Viruses at Egyptian National Research Centre and were grown in Dulbecco’s Modified Eagle’s Medium (DMEM) (DMEM; BioWhittaker, Walkersville, MD, USA) supplemented with fetal bovine serum (FBS) (10%) (Gibco-BRL; New York, USA) and penicillin/streptomycin (pen/strep) antibiotic/antimycotic mixture (2%) (GIBCO-BRL; New York, USA) and cultured under the optimum growth conditions; 37 °C/ 5% CO_2_/humified conditions.

Both the HPAIV A/chicken/Egypt/N12640A/2016(H5N1)^22^ and the seasonal influenza A/Egypt/NRC098/2019(H1N1) (GISAID ID: EPI_ISL_12995118) were provided by the Egyptian Center of Scientific Excellence for Influenza Viruses, National Research Center, Egypt and inoculated either in MDCK cells and/or specific pathogen-free (SPF) embryonated chicken eggs to propagate them as described earlier^22,23^.

### Compounds under investigation

**Figure 1:**
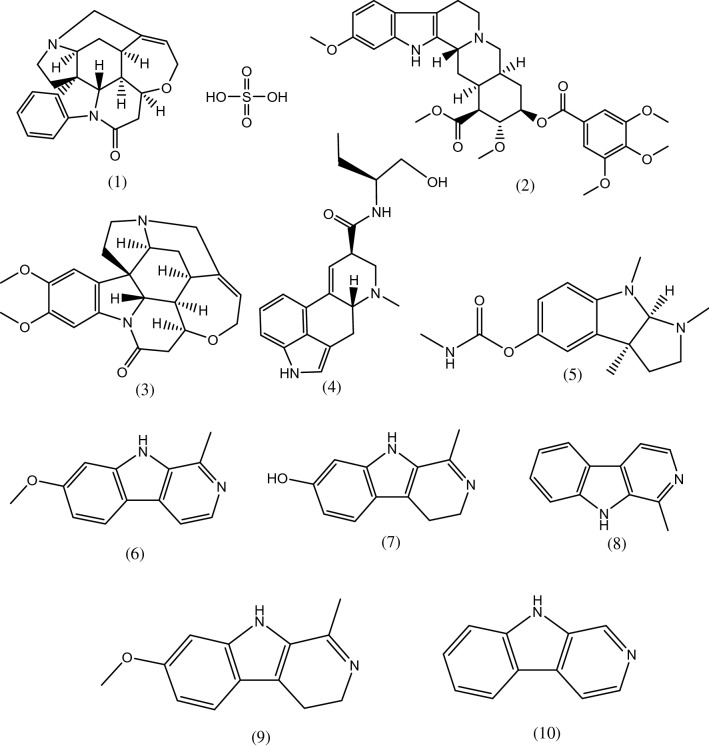
The molecular structures of the studied compounds. (1) Strychnine sulfate, (2) Reserpine, (3) Brucine, (4) Methyl ergometrine, (5) Eserine, (6) Harmine, (7) Harmalol, (8) Harmane, (9) Harmaline, (10) Norharmane.

The tested compounds used in our investigation are listed, in detail (Table 1). Strychnine sulfate, reserpine, brucine, eserine, harmine, and norharmane were purchased from Sigma-Aldrich (Sigma-Aldrich, Germany). Harmane, harmaline, and harmalol were purchased from the Molekula group (Molekula, Germany). Methyl ergometrine was obtained from Novartis (Al-Amiria District, Cairo). Their documented biological activities/chemical nucleus served as the basis for their selection to be examined for their antiviral potential against IAVs. The molecular structures of the studied compounds are shown (Fig. 1).

**Table 1:** The tested indole alkaloid and β-carboline indole alkaloid compounds.

Compound	Class	Biological activities	Viruses	Citation
Strychnine Sulfate	Indole Alkaloids	Antitumor	N/A	^24^
Reserpine	Indole Alkaloids	Anti-inflammatory, antihypertensive, and antiviral activities	SARS-CoV	^15,25,26^
Brucine	Indole Alkaloids	Anticarcinogenic, neurotoxic, anti-inflammatory, and analgesic	N/A	^27–29^
Methyl ergometrine	Indole Alkaloids	Antihemorrhagic	N/A	^30^
Eserine	Indole Alkaloids	Anticholinesterase and antiviral	SARS-CoV-2	^14,31^
Harmine	β-carboline Indole Alkaloids	Anticarcinogenic, antiviral antifungal, antimicrobial, antiplasmodial, and antioxidant	HSV-2, DENV-2, and EV-71	^17–20^
Harmalol	β-carboline Indole Alkaloids	Anticarcinogenic and antifungal	N/A	^32,33^
Harmane	β-carboline Indole Alkaloids	Antianxiety, antidepressant, antidiabetic, antioxidant, antiparasitic and antiviral	HSV-1 & HSV-2	^34–36^
Harmaline	β-carboline Indole Alkaloids	Antioxidant and anti-tumor	N/A	^37,38^
Norharmane	β-carboline Indole Alkaloids	Anticancer and antibacterial	N/A	^39,40^

### Virus titration

#### Median tissue culture infectious dose (TCID_50_) method

To determine the viral dilutions that can infect 50% of the MDCK cell line, we conducted the TCID_50_ method according to Reed and Muench method (1938)^41^ method. Briefly, ten-fold serial viral dilutions, in triplicates, were allowed to infect MDCK cell monolayers and incubated at 37 °C in a humidified 5% CO_2_ incubator for 72 h.

After incubation, plates were decontaminated and cells were fixed using a 10% paraformaldehyde solution. An aliquot of 100 µl crystal violet stain (0.1% in methanol) was added to each well and allowed to incubate at room temperature for 10 min. Plates were next thoroughly washed with water to remove excess staining material and dried overnight. Once dried, plates were assessed for cytopathic effect (CPE) in each column. The final titer was calculated using the Reed–Muench Method^41^.

#### Plaque infectivity assay (PIA)

To define the countable viral titer of IAVs and define their stock titers as plaque-forming units (PFU)/ml, PIA was performed as previously described^42^ with minor modifications. Briefly, the 80–90% confluent MDCK cell monolayers (precultured at a density of 1.2 × 10^6^ cells/well in 6 well plate) were washed with 1X phosphate buffer saline (1xPBS) and infected with 100 µl/well of the ten-fold serial dilutions for each virus in 1xDMEM (supplemented with 4% bovine serum albumin (BSA) (Gibco-BRL; New York, USA), 2% pen/strep mixture and 1 mg/mL of L‐1‐tosyl‐ amido‐2‐phenylethyl chloromethyl ketone (TPCK)-treated trypsin). The plates were incubated for 1 h in a humified condition with 5% CO_2_ and 37 °C to allow for viral adsorption. The plates were gently shaken during the incubation period at intervals of 15 min. The inocula were then removed and the plates were overlayed with 2% agarose/2xDMEM overlayer, then the plates were incubated for 72 h at 37 °C with 5% CO_2_ in a humidified condition. The plates underwent fixation with 10% formaldehyde and were further stained with 0.1% crystal violet (CV) solution to visualize the viral plaques. The following formula was used to determine the viral titer for each virus:

$$ {\text{Plaque}}\;{\text{forming}}\;{\text{unit}}\;\frac{{{\text{PFU}}}}{{{\text{ml}}}} = {\text{Number}}\;{\text{of}}\;{\text{plaques}}\;*\;{\text{Reciprocal}}\;{\text{of}}\;{\text{virus}}\;{\text{dilution}}\;*\;{\text{Dilution}}\;{\text{factor}}\;({\text{to}}\;1\;{\text{ml}}) $$

### Cytotoxicity and antiviral assays

The crystal violet assay was employed as previously described to determine the half maximum cytotoxic concentration 50 (CC_50_) on MDCK cells and the half maximal inhibitory concentrations 50 (IC_50_) for each compound^43–45^. Briefly, MDCK cells were cultivated into cell culture plates (100 µl/well at a density of 3 × 10^5^ cells/mL) and incubated for 24 h under the optimal previously described growth conditions. Next, the plates were washed with sterile 1xPBS, and successive ten-fold serial dilutions of the investigated natural-based compounds were added to the cultured wells in triplicate including untreated cell control wells. The plates were then incubated at 37 °C / 5% CO_2_ in humidified conditions for 3 days to assess the CC_50_ for each compound. After the incubation period, the cell monolayers were fixed with 10% formaldehyde and then stained with 0.1% crystal violet. The crystal violet stain in dried plates was then dissolved by adding absolute methanol. The optical density (OD) was measured using an ELISA plate reader at a wavelength of 570 nm.

The IC_50_ was performed as previously described^44^. In 96-well tissue culture plates, MDCK cells were distributed in each well as described previously and incubated overnight at a humidified 37 °C incubator under 5% CO_2_ condition. The cell monolayers were then washed once with 1 × PBS and subjected to virus adsorption (100 TCID_50_/ml) for 1 h at room temperature (RT). The cell monolayers were further overlaid with 100 μl of 1xDMEM containing varying safe concentrations of the test compounds, including untreated cell control wells and virus-infected untreated cells as a virus control for normalization. Following incubation at 37 °C in a 5% CO_2_ incubator for 72 h, the cells were fixed with 100 μl of 4% paraformaldehyde for 20 min and stained with 0.1% crystal violet in distilled water for 15 min at RT. The crystal violet dye was then dissolved using 100 μl absolute methanol per well and the optical density of the color is measured at 570 nm using Anthos Zenyth 200rt plate reader (Anthos Labtec Instruments, Heerhugowaard, Netherlands). The IC_50_ of the compound is that required to reduce the virus-induced cytopathic effect (CPE) by 50%, relative to the virus control.

### Plaque reduction assay (PRA)

To further verify the anti-influenza potential of the most promising candidates based on their IC_50_ values, the PRA was conducted according to Mostafa and coworkers^44^ with minor modifications. Briefly, the MDCK cells were cultured in 6 well plate at a density of 1.2 × 10^6^ cells/well under the optimum growth conditions; 37 °C in humified 5% CO_2_ conditions. The non-toxic concentrations of each compound were mixed with countable virus dilution (A/H1N1 or A/H5N1) in 1xDMEM supplemented with 4% BSA, 2% pen/strep mixture, and TPCK-treated trypsin and incubated at room temperature for 1 h. To allow viral adsorption, the mixture was added to the MDCK cell monolayer (80–90% confluency) under optimal growth conditions and incubated for 1 h at 37 °C in a humidified 5% CO_2_ incubator. The inocula were then aspirated and 2% agarose/2 × DMEM overlayers were added. The plates were incubated at 37 °C in a humidified 5% CO_2_ incubator for 72 h. The cell monolayers in plates were then fixed with 10% formaldehyde and further stained with 0.1% crystal violet (CV) solution to visualize the viral plaques. The percent of viral reduction was calculated using the following equation:

$$ \user2{Plaque}\;\user2{reduction}\;(\% ) = \frac{{{\mathbf{Count}}\;{\mathbf{of}}\;{\mathbf{untreated}}\;{\mathbf{virus}}\;({\mathbf{control}})\; - \;{\mathbf{Count}}\;{\mathbf{of}}\;{\mathbf{treated}}\;{\mathbf{virus}}}}{{{\mathbf{Count}}\;{\mathbf{of}}\;{\mathbf{untreated}}\;{\mathbf{virus}}\;({\mathbf{control}})}}\; \times \;{\mathbf{100}} $$

### Possible stage(s) of antiviral action

To define the possible stage(s) of antiviral action for each anti-influenza candidate hitting high selectivity index, the plaque reduction assay^46^ was carried out with some modifications to discover the critical step at which the tested compound work against the influenza A/H5N1 virus. According to Mostafa and coworkers^44^, the compound exerts its antiviral effect using the following mechanisms: (1) hindering the viral multiplication inside the host cell, (2) hindering the viral adsorption onto the host cell, or (3) direct viricidal effect against the virus (a cell-free mechanism).

#### Viral replication interference

The viral dilutions of influenza A/H5N1 virus were allowed to infect the MDCK cells that were previously cultured at a density of 1.2 × 10^6^ cells/well in 6 well plate for 24 h under optimal conditions. Each plate contains cell and virus control wells to ensure the validity of the assay and to calculate the percent of viral inhibition following treatment, respectively. The plates were then then incubated at 37 °C in a humidified 5% CO_2_ incubator for 1 h. The cell monolayers were then washed with 1xPBS to remove the residues of the infection process. Subsequently, the different predetermined non-cytotoxic concentrations of each compound were applied, and the plates underwent a second incubation period at 37 °C in a humidified 5% CO_2_ incubator for 1 h. Another washing step was applied and the 2% agarose overlayers were added then the plates were incubated at 37 °C in a humidified 5% CO_2_ incubator for 72 h. The cell monolayers were then fixed and stained, visualized using 0.1% crystal violet solution as described previously in the plaque infectivity assay.

#### Viral adsorption interference

A range of non-cytotoxic concentrations was applied to the precultured MDCK cells in 6 well plates (1.2 × 10^6^ cells/well). Each plate contains cell and virus control wells to ensure the validity of the assay and to calculate the percent of viral inhibition following treatment, respectively. The plates were then incubated in the refrigerator (4 °C) for 1 h to allow chemical adsorption onto cell receptors without active penetration. The plates were then washed with 1xPBS to remove the residual compounds. Subsequently, the countable viral dilution of influenza A/H5N1 virus was applied to allow viral adsorption/infection and another incubation period was employed at 37 °C in a humidified 5% CO_2_ incubator for 1 h. The cell monolayers were then washed with 1xPBS to remove the residual virus and overlayed with 2%Agarose/2xDMEM overlay and incubated at 37 °C in a humidified 5% CO_2_ incubator for 72 h. The cell monolayers were then fixed and stained, visualized using 0.1% crystal violet solution as described previously in the plaque infectivity assay.

#### Viricidal effect

A simple plaque reduction assay was performed where effective concentrations of the compounds were mixed with concentrated influenza A/H5N1 virus (3–4 folds higher than the countable virus dilution). The virus/compound mixture was then incubated at room temperature for 1 h. Subsequently, ten-fold serial dilutions of the virus/compound mixture (3 or 4 times) were performed to reach a countable viral titer. The mixture dilution with countable viral titer was then applied to the MDCK monolayers (1.2 × 10^6^ cells/well) including cell and virus control wells. The plates were then incubated at 37 °C in a humidified 5% CO_2_ incubator for 1 h. To remove the remains of the mixture, cell monolayers were washed with 1xPBS and overlayed with 2% agarose/2xDMEM, and incubated at 37 °C in a humidified 5% CO_2_ incubator for 72 h. The cell monolayers were then fixed and stained, visualized using 0.1% crystal violet solution as described previously in the plaque infectivity assay.

### Cheminformatics studies

#### Molecular docking

The X-ray crystal structure coordinate of (PDB ID: 6hp0^46^ and PDB: ID:6bkk^47,48^) were retrieved from PDB with their co-crystallized bound ligands. The study is represented in detail against PDB ID: 6hp0). The docking study was performed using openEye scientific software version 2.2.5 (SantaFe, NM (USA), http://www.eyesopen.com), academic license (The Laboratory of Yaseen A. M. Mohamed Elshaier. A virtual library of the synthesized compounds was used, and their energies were minimized using the MMFF94 force field, followed by the generation of multi-conformers using the OMEGA application. The library was compiled in one file by Omega. The target proteins were retrieved from PDB and the created receptor was operated by the OeDocking application. Both the ligand input file and the receptor input file were subjected to FRED to implement the molecular docking study. Multiple scoring functions were engaged to predict the energy profile of the ligand-receptor complex. The vida application was used as a visualization method. The dimension for created box of receptors were as follow: Box volume: 5544 Å, dimension 21.00 Å × 18.00 Å × 14.67 Å.

#### Physiochemical parameter and lipophilicity calculations

Compound parameters, including clogP, were calculated by using the free-access website http://www.eyesopen.com).

### Biosafety and biosecurity

All experiments with infectious viruses were performed according to Egyptian regulations for the propagation of influenza viruses. The low pathogenic seasonal IAV and the highly pathogenic AIV were handled in biosafety level 2 (BSL-2) and 3 (BSL-3) conditions in two separate laboratories, respectively, approved for such use by the local authorities.

## Results

### Cytotoxicity and viral inhibitory activity of the screened alkaloid compounds

**Figure 2:**
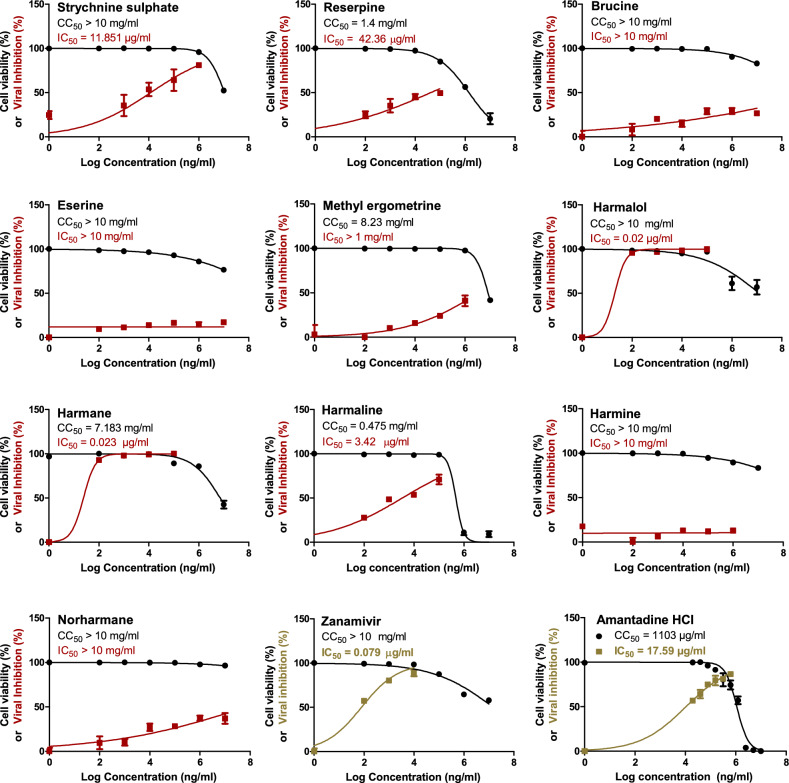
The cytotoxic and anti-H5N1 effects of the screened compounds in the MDCK cell line. By employing the crystal violet assay and nonlinear regression analysis of the GraphPad Prism program (version 5.01) by plotting log inhibitor versus normalized response (variable slope), the CC_50_ and IC_50_ of the examined indole and β-carboline indole alkaloids were determined.

The assessment of cytotoxicity of the tested indole and β-carboline indole alkaloids (Table 1) is a critical step for further evaluating their anti-influenza potential activity. The cytotoxicity of the screened alkaloid compounds was evaluated in MDCK cells (Fig. 2) and they all showed a wide range of safe, non-toxic concentrations (from 1 ng/ml to 10 mg/ml).

Thereafter, the anti-influenza potential activity of each compound was investigated at non-cytotoxic concentrations against HPAIV A/H5N1, compared to the reference anti-influenza neuraminidase inhibitor zanamivir and the M2-proton channel blocker amantadine. Remarkably, the indole alkaloid; Strychnine sulfate demonstrated a significant effect with an IC_50_ value of 11.851 µg/ml, and in the same context, the β-carboline indole alkaloids; Harmalol, Harmane and Harmaline exhibited strong anti-influenza effects with the IC_50_ values of 0.02, 0.023 and 3.42 µg/ml, respectively, with unexpected very high selectivity indices (SIs) (Fig. 2 and Table 2). On the same hand, as compared to the drug controls, Reserpine elucidated a moderate anti-influenza activity with an IC_50_ value of 42.36 µg/ml. Unlikely, Brucine, Eserine, Methyl ergometrine, Harmine, and Norharmane proved poor or no antiviral potential activity against the avian influenza A/H5N1 virus.

**Table 2:** Selectivity indices “SIs” for the tested compounds against avian influenza A/H5N1 and human influenza A/H1N1 virus in the MDCK cell line.

Phytochemical compound	Chemical classification	IAV subtype	CC_50_ (mg/ml)	IC_50_ (µg/ml)	Selectivity index (SI)
Strychnine Sulfate	Indole Alkaloids	H5N1	> 10	11.851	> 843
		H1N1		0.06	> 1.67*10^5^
Reserpine		H5N1	1.4	42.36	> 33
		H1N1		ND	N/A
Brucine		H5N1	> 10	> 10,000	N/A
		H1N1		ND	N/A
Eserine		H5N1	> 10	> 10,000	N/A
		H1N1		ND	N/A
Ergometrine		H5N1	8.23	> 1000	N/A
		H1N1		ND	N/A
Harmalol	β-Carboline Indole Alkaloids	H5N1	> 10	0.02	> 5*10^5^
		H1N1		0.035	> 2.8*10^5^
Harmane		H5N1	7.183	0.023	> 3.12*10^5^
		H1N1		0.033	> 2.17*10^5^
Harmaline		H5N1	0.475	3.42	138
		H1N1		0.056	8482
Harmine		H5N1	> 10	> 10,000	N/A
		H1N1		ND	N/A
Norharmane		H5N1	> 10	> 10,000	N/A
		H1N1		N/A	N/A
Zanamivir	NAI (drug control)	H5N1	> 10	0.079	> 1.26*10^5^
		H1N1		0.291	> 3.4*10^4^
Amantadine HCl	M2-channel blocker	H5N1	1.103	17.59	62.71
		H1N1		14.86	74.32

**Figure 3:**
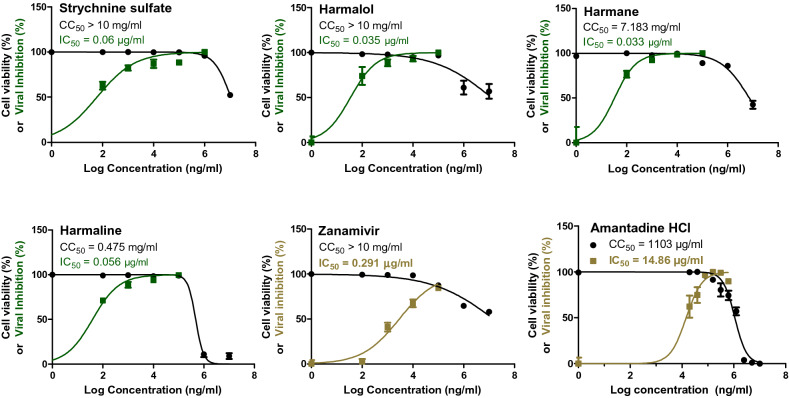
The cytotoxic and anti-H1N1 effects of the screened compounds in the MDCK cell line. By employing the crystal violet assay and nonlinear regression analysis of the GraphPad Prism program (version 5.01) by plotting log inhibitor versus normalized response (variable slope), the CC_50_ and IC_50_ of the most promising indole and β-carboline indole alkaloids were computed.

To confirm that the obtained anti-influenza activities of Strychnine sulfate, Harmalol, Harmane, and Harmaline are not strain-specific, their anti-influenza activity was further investigated against human influenza A/H1N1 virus and compared to the anti-influenza zanamivir and amantadine drug controls. Consistently, Strychnine sulfate, Harmalol, Harmane, and Harmaline exerted potent anti-influenza activity against influenza A/H1N1 virus with IC_50_ values of 0.06, 0.035, 0.033, and 0.056 µg/ml respectively (Fig. 3).

### Viral plaque reduction of the investigated compounds

**Figure 4:**
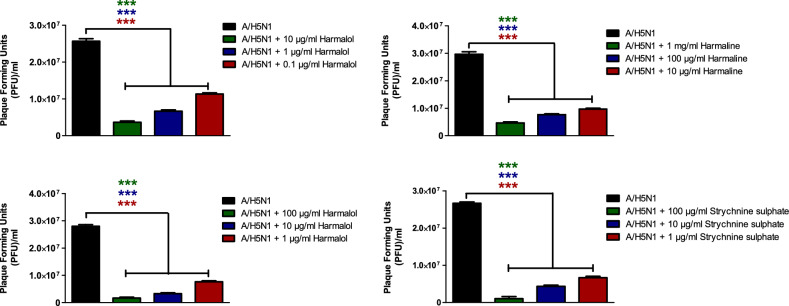
The Plaque forming units (PFU) per ml for compound-untreated influenza A/H5N1 virus control versus multiple concentrations-treated virus. Mean values of results represent the averages from three independent experiments and are presented with standard deviations (SDs) indicated by error bars. Asterisks (***) indicate a significant difference (*p* < 0.001) compared to the compound-untreated influenza A/H5N1 virus control. Statistical analysis was performed using repeated measures ANOVA, followed by Bonferroni post hoc test.

**Figure 5:**
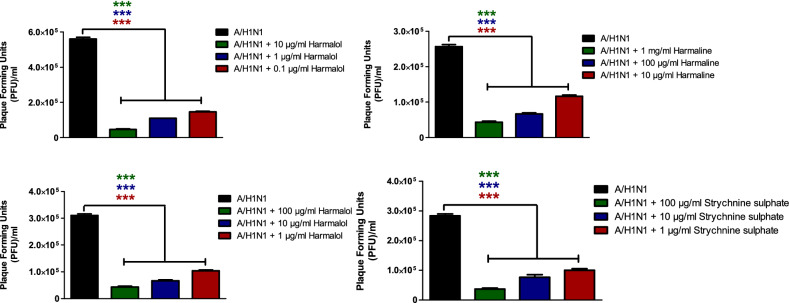
The Plaque forming units (PFU) per ml for compound-untreated influenza A/H1N1 virus control versus multiple concentrations-treated virus. Mean values of results represent the averages from three independent experiments and are presented with standard deviations (SDs) indicated by error bars. Asterisks (***) indicate a significant difference (*p* < 0.001) compared to the compound-untreated influenza A/H1N1 virus control. Statistical analysis was performed using repeated measures ANOVA, followed by Bonferroni post hoc test.

The screened alkaloids with promising anti-influenza activities as indicated with low IC_50_ values and high SI values were subjected further to plaque reduction assay to validate their anti-influenza efficacy against HPAIV A/H5N1 and seasonal influenza A/H1N1 viruses. The tested indole and β-carboline indole alkaloids demonstrated remarkable capacity to elicit viral suppression at low doses against IAVs that were used in this study (Table 3). These viral inhibitions in plaque forming units were statistically significant among all tested concentrations for each compound when compared to compound-untreated virus control for influenza A/H5N1 (Fig. 4) and A/H1N1 viruses (Fig. 5). These data are consistent with our prior findings.

**Table 3:** Viral reduction percentages following treatment of influenza A/H1N1 and A/H5N1 with a range of non-cytotoxic concentrations of indole and β-carboline indole alkaloids, as measured by plaque reduction assay.

Compound	Conc. (µg/ml)	Against influenza A/H5N1 virus			Against influenza A/H1N1 virus		
		Viral inhibition (%)			Viral inhibition (%)		
		Mean	SD	N	Mean	SD	N
Harmalol	10	86.42	2.14	3	91.81	1.01	3
	1	73.33	2.31	3	79.63	0.000	3
	0.1	54.67	2.31	3	74.27	1.02	3
Harmane	1000	84.95	1.86	3	84.62	1.01	3
	100	74.44	1.93	3	74.36	2.22	3
	10	65.48	2.06	3	55.13	2.22	3
Harmaline	100	94.25	1.99	3	86.46	1.80	3
	10	87.66	2.14	3	77.78	1.92	3
	1	72.62	2.06	3	66.67	1.86	3
Strychnine sulphate	100	96.30	3.71	3	87.36	1.99	3
	10	83.95	2.14	3	71.61	5.66	3
	1	74.36	2.22	3	65.52	3.45	3

### Stage(s) of antiviral action

To investigate the possible target(s) of the studied compounds with promising anti-influenza activities, we studied three main targets by which the compounds can affect viral infectivity and replication cycle including (1) direct viricidal action, (2) hindering the viral adsorption onto host cell’s receptor, and (3) hindering the viral replication machinery. Interestingly, the tested anti-influenza alkaloids could affect the three aforementioned compartments but in varying strengths. Interestingly, Harmalol and Harmane could block influenza A/H5N1 infection via interference with the viral replication compartment. Whereas harmaline exerted a direct cell-free viricidal effect on the viral particle. Finally, Strychnine Sulfate showed the capacity to reduce the viral titer by hindering IAV adsorption to the host cell (MDCK) receptors (Table 4).

**Table 4:** The mechanism of antiviral action for the investigated indole and β-carboline indole alkaloids represented as viral inhibition (%) in a concentration-dependent manner.

Compound	Concentration (µg/ml)	Mechanism of Action		
		Viral replication (%)	Viricidal (%)	Viral adsorption (%)
Harmalol	0.1	87.5	41.5	60.3
	1	96.9	50.9	74.5
	10	98.7	58	82.1
Harmane	10	83.3	31.8	43.4
	100	97.5	41.8	48.7
	1000	96.7	82.1	61.4
Harmaline	1	28.3	91	34
	10	40.3	96.7	46.2
	100	48.7	98.6	51.8
Strychnine Sulfate	1	19.4	29.4	90.1
	10	27.3	37	99.5
	100	42	49.2	99

### Chemoinformatic studies

#### Molecular docking study with neuraminidase (PDB ID: 6hp0)

Neuraminidase inhibitors hinder the function of the viral neuraminidase protein, preventing virus release from infected host cells to further infect new host cells and thereby freeing the virus to infect other cells in the host organism and subsequently hindering the viral replication cycle^1,49^. The main structural neuraminidase (NA) inhibitors are cyclohexane scaffold containing lipophilic moiety with modification in the geometry and size of this part^50^. Our active compounds belong to carboline skeleton in which cyclohexene ring fused with indole moiety. These similarities guided us to introduce these compounds as neuraminidase inhibitors. By comparative analysis of the docking mode and pose of our compounds to standard ligands for M2-proton channel blockers and neuraminidase inhibitors, the selected compounds revealed high similarity to the control neuraminidase inhibitors. The current antiviral drugs are likely directed to neuraminidase inhibitor analogues especially with the reported resistance of amantadine to the currently circulating IAV strains including the tested virus strain (Figs. 2 and 3). These evidences directed us to perform the docking against neuraminidase surface glycoprotein.

**Figure 6:**
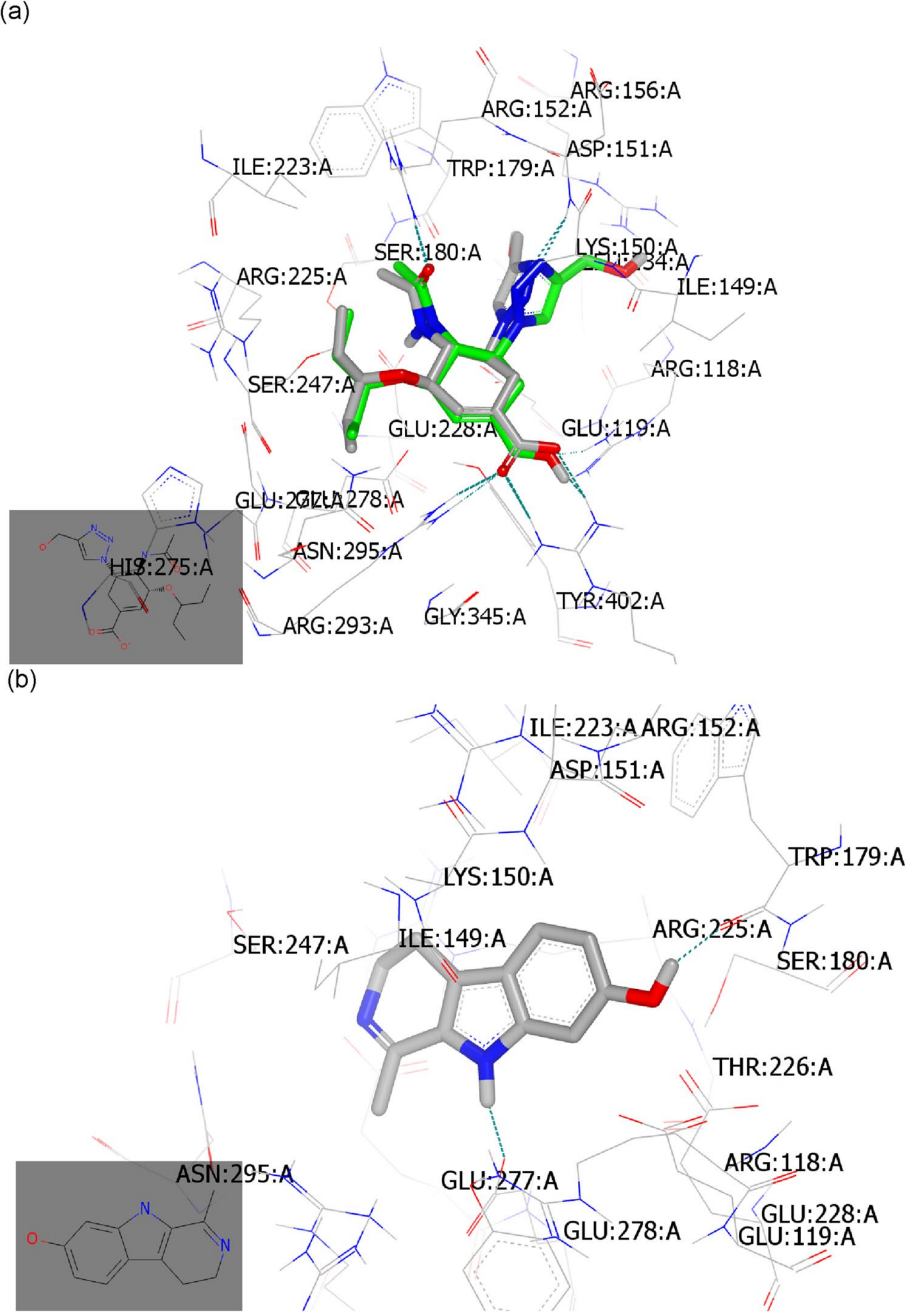

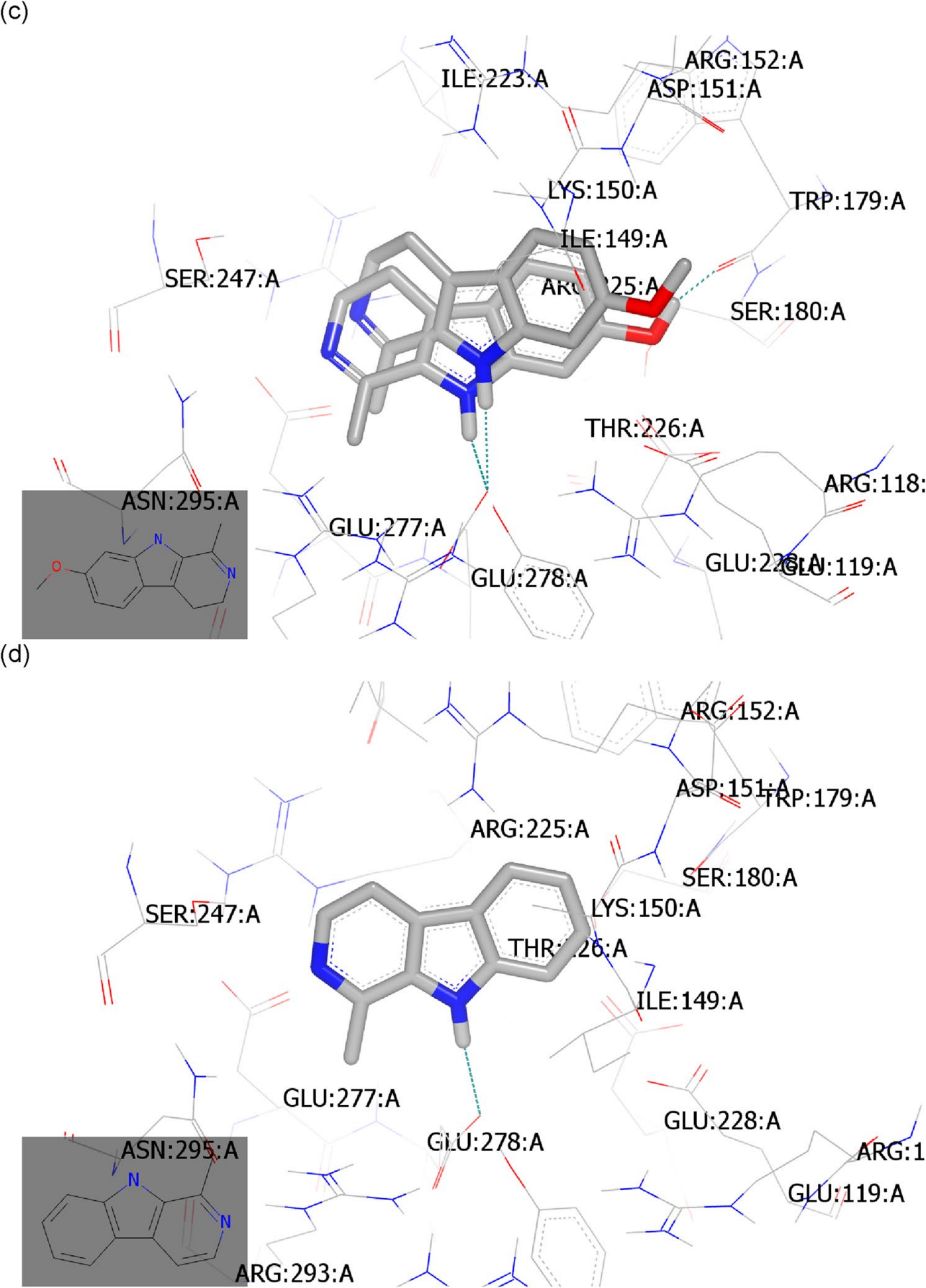
Snapshot of selected compounds represented by Vida application. (**a**) Standard ligand (grey colour) and its co-crystalized complex (green colour); (**b**) Harmalol docked with the receptor with the formation of HB (green colour); (**c**) Harmalol overlay with its methylated isomer, Harmaline; (**d**) Harmane showed complete overlay with its inactive derivative, Harmine.

To validate our docking protocol, the standard ligand was docked by OpenEye software and it was superimposed with its co-crystalized downloaded complex^51^. Both structures displayed the same binding mode and pose with consensus score 10 as the best score. They formed multiple hydrogen bonding (HB) interactions with key amino acids in the receptor active sites especially Arg: 153A, Asp:151A, Arg:293A, Arg:368A, Glu:277A, and Arg:118A. Notably, the standard ligand formed HB interactions with basic amino acids (Fig. 6a).

Harmalol forms a hydrogen bond (HB) with Trp:179A (as donor) through its OH as acceptor and with Gln: 278A through NH, (Fig. 6b) with consensus score 45. The snapshot of harmalol with its methylated isomer, harmaline represented overlay. However, harmaline was not able to form HB interaction with the receptor (Fig. 6c) with a consensus score of 63. Comparing both compounds with the inactive analog, harmine highlighted the retardation effect of methylated benzene of indole moiety. Furthermore, the potent unsubstituted derivative, harmane, showed HB with Gln:278A (Fig. 6d) with a consensus score of 56.

#### Ligand efficiency and lipophilic efficiency profile^52^

Evaluating the lipophilicity profile in the ligand-target interaction concept is an important aspect of the druggability of new bioactive candidates. Presently, rationalization of both molecular size, and lipophilicity (cLogP) with drug activities (PIC_50_) designated is essential. Ligand efficiency (LE) of bioactive compounds is assessed based on pIC_50_ in relation to the number of heavy atoms in a molecule known as non-hydrogen atoms (NHAs). LE estimates the affinity of drugs based on their size instead of considering the effectiveness or binding affinity of the whole structure. LE = ΔG ÷ NHA or **LE = (pIC**_**50**_** × 1.37) ÷ NHA**; where ΔG = Gibb's free energy, IC_50_ = half-maximal inhibitory concentration (in terms of molar concentration), and NHA = non-hydrogen atom.

The challenge in the druggability of new drug candidates is in increasing the activity while keeping lipophilicity constant to avoid any “molecular obesity” during the drug development process. The other parameter of lipophilicity is the ligand lipophilic efficiency (LLE). LLE is a way to calculate the affinity of a drug candidate’s function in its lipophilicity. LLE is the difference between the potency and ClogP according to the following equation: **LLE = pIC50- CLogP**. Table 5 illustrated selected physicochemical properties which controlled the rule of five. All compounds have a small molecular weight in the range of 182–200. Both harmalol and harmaline displayed the same number of HB as acceptor (value 3) while harmane has HB as an acceptor of value 2. Both harmane and harmaline have the same HB as a donor (value 1) while harmalol has One HB as a donor. Only harmaline has one rotatable bond. The polar surface area for harmalol, harmaline, and harmane are 48, 37, and 28 respectively. The naturally occurring indole alkaloids in (Table 4) showed LE values against H1N1 and H5N1 of 0.69–0.75. The acceptable LE value should be more than 0.3. However, all compounds revealed LEE values in the range of 6.43–7.32. An LLE value ≥ 5 is recommended for drug candidates.

**Table 5:** Physicochemical parameters and lipophilicity metrics for selected carboline alkaloids.

Compound	Mwt	NHA	ClogP	PSA	Rotatable bond	Lipinski acceptor	Lipinski donor	PIC50 H5N1	LE	LEE	PIC50 H1N1	LE	LEE
Harmalol	200	15	0.97	48	0	3	2	7.677	0.70	6.71	7.70	0.70	6.73
Harmane	182	14	2.56	28	0	2	1	7.638	0.75	5.08	7.74	0.76	5.18
Harmaline	214	16	1.71	37	1	3	1	4.552	0.39	2.84	7.70	0.70	6.74

M.wt: Molecular Weight, NHA: non-hydrogen atom known as a number of heavy atoms, Clog P: lipophilicity, IC_50_: Inhibitory Concentration, pIC_50_: ˗log IC_50_ (indicates drug potency), LE: Ligand efficiency, LLE: ligand lipophilic efficiency.

#### Structure–activity relationship (SAR)

Generally, carboline alkaloids revealed better antiviral activities than indole alkaloids except strychnine sulfate showed potent in vitro activity against H1N1 (IC_50_ = 0.06 µg/ml). Harmine and Norharmane have no effect. Harmalol, Harmane, and Harmaline illustrated equal activity against both viruses and were more potent than standard zanamivir drug. Furthermore, the free NH of the indole skeleton and low molecular weight seem important in compound activity. Among these alkaloids, harmalol has the highest PSA value and displayed the best lipophilicity metrics (LE and LEE). The compound’s geometry and aromaticity illustrated the retardation effect in compound activity especially in the case of harmaline in comparison to the inactive compound harmine.

## Discussion

Influenza is a highly transmissible respiratory disease that occasionally causes pandemics as well as seasonal epidemics. The annual seasonal viral epidemics kill up to 1 million people and infect about 10% of the human population all over the world^53^. Despite that immunization is crucial and may help to minimize the viral infection severity, it only works when the vaccine matches the target viral strains that are already in circulation^54–56^.

There are different FDA-approved and/or licensed anti-influenza agents available in the global markets, but their efficacy to control epidemic and pandemic influenza A viruses is now questionable with increased ratios of drug-resistant variants^1^. The viral resistance against the available anti-influenza medications is the result of the development of IAV strains under the selection pressure of “antigenic shift” and/or “antigenic drift”^1,57^. In this context, the decreased susceptibility to the already established antiviral medications urges the need to find new antiviral agents to combat the emerging and re-emerging flu strains.

A huge number of naturally derived, biologically active compounds containing the indole nucleus serve as therapeutics for many diseases such as cancer, diabetes, and viral and microbial diseases^58^. Hence, we sought to examine the antiviral efficacy of some indole and β-carboline indole alkaloids against seasonal influenza A/H1N1 and highly pathogenic avian influenza A/H5N1 viruses. Intriguingly, the highly promising indole alkaloid, Strychnine Sulfate, proved its ability to combat the tested avian lAV by blocking mainly the adsorption of the virus to MDCK host cells, however no documented data available regarding the antiviral and/or anti-influenza activity of strychnine sulfate.

On the same hand, the β-carboline indole alkaloids including Harmalol and Harmaline showed highly promising anti-influenza activities against influenza A/H1N1 and A/H5N1 viruses. Previous results on the total seed extract of *Peganum harmala* L. containing Harmalol, Harmine, Harmaline, and Harmane proved to exert anti-influenza activity against influenza A/Puerto Rico/8/34 (H1N1; PR8) virus with an IC_50_ value of 9.87 µg/ml for the crude extract and 5.8 µg/ml for the total purified alkaloids^59,60^. However, rare or no information is available about the anti-influenza activities of Harmalol or Harmaline as purified compounds.

On the other hand, Harmane was shown to elucidate its capacity to counteract both seasonal human A/H1N1 virus and avian influenza A/H5N1 virus. In addition to the diverse range of Harmane, the purified Harmane from two different Simira plants, a genus of plants in the family Rubiaceae, showed antiviral potential against two different types of herpes simplex viruses (HSV-1 and HSV-2) with EC_50_ (half maximal effective concentration) values of 4.90 µg/ml for HSV-1 and 71.8 µg/ml for HSV-2^35^. Regarding the influenza viruses, no evident proof of the anti-influenza properties of pure Harmane was found in the literature. however *P. harmala* seeds extract undergone previous in vivo study against IAV^60^.

As to their safety, strychnine is known to cause muscle contraction and convulsions that can lead to respiratory paralysis at high dose of 1.5 mg/kg^61^. In the same line, alkaloids from *P. harmala* seeds extract were reported to be toxic at higher doses ranging from 38 to 200 mg/kg in experimental animals^62^. This confirm that these studied alkaloids are highly tolerated in vivo due to previous studies, but they should be further confirmed in vivo to prove their safety and efficacy.

On the contrary, Reserpine was documented to have a robust antiviral activity against severe acute respiratory syndrome coronavirus (SARS-CoV) with an EC_50_ value of 3.4 µM^15^ whilst in our findings, it proved moderate anti-influenza potential against the human influenza A/H1N1 virus. Similarly, an in silico study for the antiviral activity of the Eserine (or Physostigmine) derivatives against pandemic SARS-CoV-2 proved their ability to combat the coronavirus action^14^. However, the purified compound of the Eserine used in this study showed no antiviral activity against the human influenza A/H1N1 virus. Moreover, documented data regarding the antiviral activities of the β-carboline indole alkaloid, Harmine, proved its extensive use as an antiviral agent against HSV-2 with an EC_50_ value of 1.47 µM^17^. However, 9 N methylharmine, a Harmine derivative, proved to inhibit the DENV-2 (dengue virus-2) with an EC_50_ value of 3.2 ± 0.6 µM^18^. Furthermore, Harmine exerted an antiviral property against EV-71 (Enterovirus-71) infection with an EC_50_ value of and 20 µM^19^. Nevertheless, our study proved no anti-influenza activity of the purified β-carboline indole alkaloid, Harmine, against avian influenza A/H5N1 virus.

Moreover, the four potent anti-influenza candidates can affect the viral replication cycle at multiple stages with variable inhibition levels, Strychnine Sulfate can predominately impair viral adsorption, while Harmalol and Harmane can affect mainly by interfering with the viral replication and finally Harmaline that affects the virus predominately in a cell-free status (direct virucidal effect). Nevertheless, this stage of the mechanism study is a preliminary overview to highlight the possible stage(s) in the viral replication cycle that may get affected during treatment with the investigated compound. To this point, further molecular investigations are demanded to specifically define the exact viral and cellular targets of the investigated compounds.

Conclusively, this study highlighted some alkaloids with robust anti-influenza activity. By applying the chemoinformatic studies, we found that harmalol displayed the best druggability parameters, especially LE and LLE) against both viruses. We, therefore, recommend that the antiviral activities of the studied effective alkaloids have to be widely studied including in vivo studies.

## Conclusion

Alkaloids are natural secondary metabolites of plant origin that increasingly attract attention due to their numerous pharmacological actions including antiviral activities. This study could successfully investigate the anti-influenza efficacy of some distinct biologically active indole and β-carboline (βC) indole alkaloids against seasonal human-type IAV and avian-type IAV. Interestingly, β-carboline (βC) indole alkaloids displayed robust anti-influenza activities against the predefined strains via multiple modes of action. Cheminformatics studies and lipophilicity metrics for active compounds illuminate our direction for further studies for preclinical and clinical phases as antiviral drug candidates.

## Data Availability

All data generated or analyzed during this study are included in this published article.

## Abbreviations

IC_50_: Half maximal inhibitory concentration
CC_50_: Half maximal cytotoxic concentration
SI: Selectivity Index
MDCK: Madin Darby Canine Kidney
HPAIV: Highly pathogenic avian influenza virus
SARS-CoV-2: Severe acute respiratory syndrome coronavirus 2
LE: Ligand efficiency
LLE: Ligand lipophilic efficiency
NHA: Non-hydrogen atom
SAR: Structure–activity relationship
